# Plasma proteomics reveals crosstalk between lipid metabolism and immunity in dairy cows receiving essential fatty acids and conjugated linoleic acid

**DOI:** 10.1038/s41598-022-09437-w

**Published:** 2022-04-05

**Authors:** Arash Veshkini, Harald M. Hammon, Laura Vogel, Didier Viala, Mylène Delosière, Arnulf Tröscher, Sébastien Déjean, Fabrizio Ceciliani, Helga Sauerwein, Muriel Bonnet

**Affiliations:** 1 Institute of Animal Science, Physiology Unit, University of Bonn, Bonn, Germany; 2 Research Institute for Farm Animal Biology (FBN), 18196 Dummerstorf, Germany; 3 INRAE, Université Clermont Auvergne, VetAgro Sup, UMR Herbivores, 63122 Saint-Genès-Champanelle, France; 4 Department of Veterinary Medicine, Università degli Studi di Milano, Lodi, Italy; 5 Metabolomic and Proteomic Exploration Facility (PFEM), INRAE, 63122 Saint-Genès-Champanelle, France; 6 BASF SE, 68623 Lampertheim, Germany; 7 Institut de Mathématiques de Toulouse, UMR5219, Université de Toulouse, CNRS, UPS, 31062 Toulouse, France

**Keywords:** Computational biology and bioinformatics, Molecular biology, Physiology, Zoology

## Abstract

Essential fatty acids (EFA) and conjugated linoleic acids (CLA) are unsaturated fatty acids with immune-modulatory effects, yet their synergistic effect is poorly understood in dairy cows. This study aimed at identifying differentially abundant proteins (DAP) and their associated pathways in dairy cows supplied with a combination of EFA and CLA during the transition from antepartum (AP) to early postpartum (PP). Sixteen Holstein cows were abomasally infused with coconut oil as a control (CTRL) or a mixture of EFA (linseed + safflower oil) and CLA (Lutalin, BASF) (EFA + CLA) from − 63 to + 63 days relative to parturition. Label-free quantitative proteomics was performed on plasma samples collected at days − 21, + 1, + 28, and + 63. During the transition time, DAP, consisting of a cluster of apolipoproteins (APO), including APOE, APOH, and APOB, along with a cluster of immune-related proteins, were related to complement and coagulation cascades, inflammatory response, and cholesterol metabolism. In response to EFA + CLA, specific APO comprising APOC3, APOA1, APOA4, and APOC4 were increased in a time-dependent manner; they were linked to triglyceride-enriched lipoprotein metabolisms and immune function. Altogether, these results provide new insights into metabolic and immune adaptation and crosstalk between them in transition dairy cows divergent in EFA + CLA status.

## Introduction

Dairy cows regardless of health status undergo a state of non-pathogenic inflammation during the transition from late pregnancy to early lactation (periparturient period), which consequently activates the immune system^1^. Recent evidence suggested that systemic inflammation may further challenge nutrient homeostasis mechanisms, with consequences that may result in decreased feed intake, increased non-esterified fatty acids (NEFA), hyperketonemia, and disorders/diseases such as hepatic steatosis^1^. Supplementing specific fatty acids (FA) with immunomodulatory properties could be a natural strategy to tolerate immune activation and prevent negative outcomes in dairy cows around parturition^2–4^.

Essential FA (EFA) including linoleic acid (LA, 18:2 n-6) and α-linolenic acid (ALA, 18:3 n-3), along with the stereo-isomers of LA, named as conjugated linoleic acid (CLA), are involved in numerous metabolic pathways such as lipogenesis, inflammation, modulation of immune functions, and gene expression regulation^4,5^. It has recently been reported that the combination of EFA and CLA supplementation during the periparturient period improves dairy cows' energy balance by inducing milk fat depression^6^ (MFD) and, as shown by the changes of specific markers, positively affects immune^2^ and metabolic homeostasis^7,8^. However, the exact underlying mechanisms by which EFA and CLA impact the metabolism of dairy cows largely remained a “black box”^3^.

Plasma is a pool with a high dynamic range of proteins, secreted by all body tissues the most active being the liver, reflecting an organism's metabolic or physiological status^9^. There is limited data on plasma proteins linked to nutritional modifications, particularly during the periparturient period, probably because the large-scale description of plasma protein pool faces methodological challenges^10^. Proteomics approaches may overcome this issue by providing high accuracy for understanding the proteome shifts in different physiological statuses and under different treatments in dairy cows, as recently described^11–13^.

Given this background, we aimed to gain insight into the plasma proteome of dairy cows supplied or not with combined EFA and CLA during the periparturient period. Longitudinal label-free quantitative proteomics was performed on plasma samples selected from a previous study according to plasma metabolite results^6^. To the best of our knowledge, this is the first report in dairy cows using longitudinal plasma proteomics to study in vivo metabolic and immune adaptation around parturition and its interaction with infused FA.

## Results

At 1% peptide false discovery rate (FDR), a total of 241 unique proteins with at least two unique peptides were identified in plasma at each individual time-point (Supplementary S1, Supplementary Table S1). Of these, 160 belonged to different classes including 41 modifying enzymes (PC00260), 36 protein-binding activity modulators (PC00095), 28 metabolite interconversion enzymes (PC00262), 21 transfer/carrier proteins (PC00219), 9 defense/immunity proteins (PC00090), 9 structural proteins (PC00211), 9 extracellular matrix proteins (PC00102), and 7 intercellular signal molecules (PC00207) (obtained under PANTHER database, http://pantherdb.org/) (Supplementary S1, Supplementary Table S2).

### Differential plasma proteome during the antepartum and postpartum period

**Figure 1:**
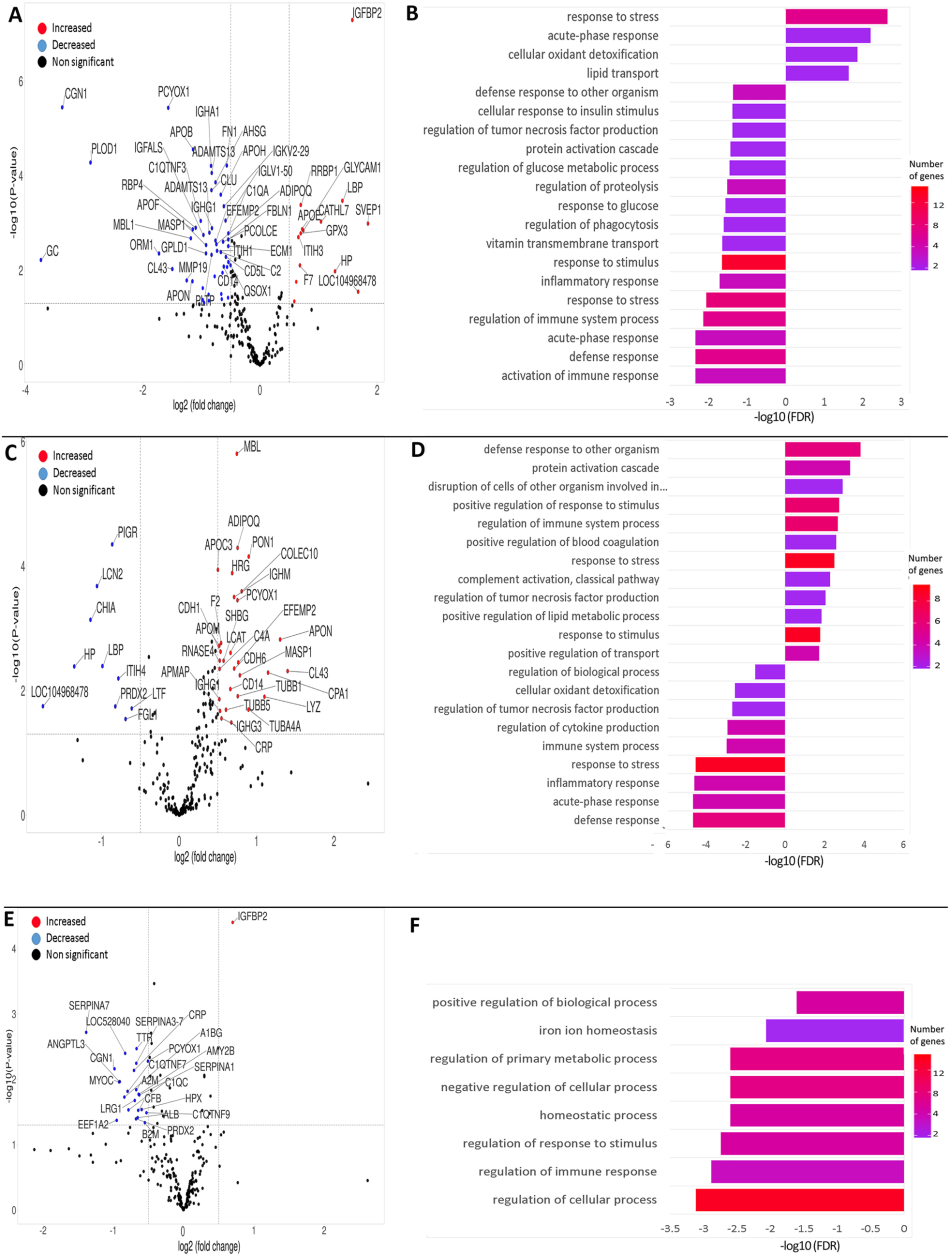
(**A**) Volcano plot representing differentially abundant proteins (DAP) between day − 21 and + 1 relative to parturition; increased (red dots in top right) and decreased (blue dots in top left) proteins in d + 1/d − 21 are highlighted (P < 0.05 and log2 fold change (FC) > 1.3). (**B**) Biological Process for the DAP Proteins after semantic synthesis by Revigo; bars indicate proportional to the false discovery rate (FDR) adjusted P-value, the intensity of color bars is gene count (GC) represents the amount of differentially abundant proteins enriched in the pathway. (**C**) Volcano plot representing DAP between day + 1 and + 28 postpartum. (**D**) Biological Process for the DAP between day + 1 and + 28 postpartum. (**E**) Volcano plot representing DAP between day + 28 and + 63 postpartum. (**F**) Biological Process for the DAP between day + 28 and + 63 postpartum (For the high quality figure, the reader is referred to the web version of this article).

From day 21 antepartum (AP) to day 1 postpartum (PP), the relative abundance of 14 proteins was increased, and of 49 proteins decreased (Log2 fold change range from − 4 to + 2, p-value ≤ 0.05) in the plasma regardless of treatment (Fig. 1A, Supplementary S2, Supplementary Table S3). The KEGG analysis mapped differentially abundant proteins (DAP) to cholesterol metabolism and complement and coagulation cascades pathways. Underabundant proteins were annotated by biological process (BP) gene ontology (GO) terms mainly related to immune regulation and glucose homeostasis, particularly through activation of immune response GO:0002253, acute-phase response GO:0006953, inflammatory response GO:0006954, complement activation GO:0006958, regulation of glucose metabolic process GO:0010906, regulation of tumor necrosis factor production GO:0032680, cellular response to insulin stimulus GO:0032869 (Fig. 1B). Overabundant proteins were annotated by BP GO terms including, response to stress GO:0006950, acute-phase response GO:0006953, cellular oxidant detoxification GO:0098869, and lipid transport GO:0006869 (Fig. 1B). The DAP were assigned to the extracellular region and lipoprotein particles based on the cellular component (CC) GO category (Supplementary S2, Supplementary Tables S4, S5, S6, S7, S8).

From day + 1 to day + 28 PP, 29 proteins were over- and 10 proteins were underabundant. Over and under-abundant proteins are displayed in the Volcano plot (Fig. 1C, Supplementary S3, Supplementary Table S9). The DAP were mapped to the phagosome, NF-kappa B signaling pathway, complement and coagulation cascades, and toll-like receptor signaling pathway using the KEGG pathway analysis (Supplementary S3, Supplementary Table S10). The GO analysis showed enrichment of similar BP terms by both up- and downregulated proteins, including response to stress GO:0006950, regulation of cytokine production GO:0001817, inflammatory response GO:0006954, and cellular oxidant detoxification GO:0098869 (Fig. 1D) (Supplementary S3, Supplementary Tables S11, S12, S13, S14).

Twenty-five proteins, including one over and 24 under-abundant ones, were identified as DAP in d + 63 over day + 28 (Fig. 1E, Supplementary S4, Supplementary Table S15). In total, three KEGG pathways, including complement and coagulation cascades, S*taphylococcus aureus* infection, and thyroid hormone synthesis, have annotated the DAP. Insulin-like growth factor-binding protein 2 (IGFBP2) as the only over-abundant protein was not annotated by a GO term, but under-abundant proteins were annotated by GO BP terms related to the regulation of immune response GO:0050776, regulation of response to stimulus GO:0048583, homeostatic process GO:0042592, GO:0010951 negative regulation of endopeptidase activity and regulation of primary metabolic process GO:0080090 (Fig. 1F) (Supplementary S4, Supplementary Tables S16, S17, S18).

### Differential plasma proteome between EFA + CLA and control groups

#### Overlap between differentially abundant proteins in response to the fatty acid treatment at different timepoints

**Figure 2:**
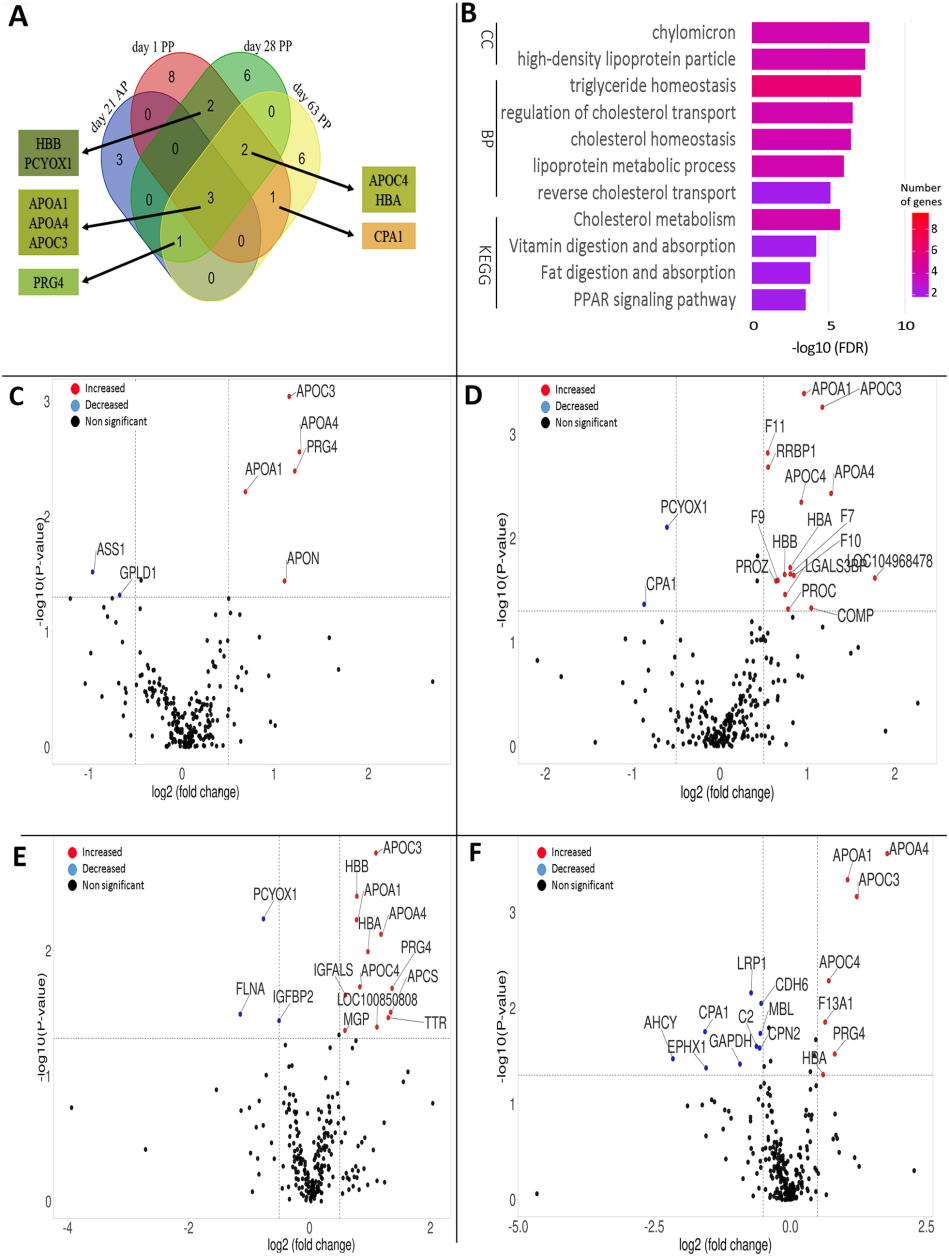
(**A**) Venn diagram representing the overlap between differentially abundant proteins (DAP) in response to EFA + CLA supplementation identified at day − 21, + 1, + 28, and + 63 relative to parturition. (**B**) KEGG pathways map^14^, biological process (BP), and cellular component (CC) gene ontology terms of DAP after semantic synthesis by Revigo (overlapping pathways). Bars represents the − log10 (adjusted P-value); the intensity of color bars is gene count (GC) represents the amount of differentially abundant proteins enriched in the pathway. (**C**) Volcano plot representing DAP between Control (CTRL) and essential fatty acid (EFA) + conjugated linoleic acid (CLA) in day 21 antepartum; increased (red dots in the top left) and decreased (blue dots in the top left) proteins in EFA + CLA group are highlighted (P < 0.05 and log2 fold change (FC) > 1.5). (**D**) Volcano plot representing DAP between CTRL and EFA + CLA in day 1 postpartum. (**E**) Volcano plot representing DAP between CTRL and EFA + CLA in day 28 postpartum. (**F**) Volcano plot representing DAP between CTRL and EFA + CLA in day 63 postpartum (For the high quality figure, the reader is referred to the web version of this article).

The Venn diagram in Fig. 2A represents the intersection of proteins identified in all timepoints in response to the EFA + CLA supplementation. The relative abundance of apolipoproteins (APO) C3 (APOC3), APOA1, and APOA4 was greater at all timepoints in response to EFA + CLA supplementation as compared to the CTRL group (“overlapping proteins”) (details in Supplementary S5, Supplementary Table S19). Also, APOC4 and hemoglobin subunit alpha (HBA) were more abundant in the EFA + CLA group in the whole PP period. These APO were mapped to KEGG pathways such as the PPAR signaling pathway, fat digestion and absorption, and cholesterol metabolism (Fig. 2B, Supplementary S6, 7, 8, and, 9, Supplementary Tables S21, S25, S29, S33). Moreover, APO proteins were annotated by enriched BP GO terms, including triglyceride homeostasis (GO:0070328), cholesterol homeostasis (GO:0042632), lipid transport (GO:0006869), plasma lipoprotein particle assembly (GO:0034377), regulation of lipoprotein lipase activity (GO:0051004), and lipoprotein metabolic process (GO:0042157) (Supplementary S6, 7, 8, and, 9, Supplementary Tables S22, S26, S30, S34). At the CC level, localization of DAP was extracellular region (GO:0005576), extracellular space (GO:0005615), high-density lipoprotein particle (GO:0034364), chylomicron (GO:0042627), and very-low-density lipoprotein particle (GO:0034361) (Supplementary S6, 7, 8, and, 9, Supplementary Tables S23, S27, S31, S35). In association with EFA + CLA supplementation, the same KEGG pathways and BP and CC GO terms were enriched in all time points (“overlapping pathways”), therefore to avoid repetition, we did not mention them in each timepoint.

#### Differentially abundant proteins in response to the fatty acid treatment at specific timepoints around parturition

On day 21 AP, seven proteins were differentially abundant between treatments (Volcano plot, Fig. 2C, Supplementary S6, Supplementary Table S20), in which, beside overlapping proteins, proteoglycan 4 (PRG4), and apoN protein (APON) were more abundant. At the same time, argininosuccinate synthase (ASS1) and phosphatidylinositol-glycan-specific phospholipase D (GPLD1) were less abundant in the EFA + CLA compared to the CTRL group. On day 21 AP, DAP were annotated only by pathways identified for the overlapping proteins (Supplementary S6, Supplementary Tables S21, S22, S23).

An overview of the proteomic variability between the treatment groups at day 1 PP is given in Fig. 2D (Supplementary S7, Supplementary Table S24). The relative abundance of 14 proteins was greater and two proteins were lower in the EFA + CLA than in the CTRL group. The overabundant proteins, were annotated by enriched BP GO terms comprised negative regulation of inflammatory response (GO:0050728), and regulation of immune system process (GO:0002682). There were no enriched pathways for the two underabundant proteins in the EFA + CLA group (Supplementary S7, Supplementary Tables S25, S26, S27).

At day 28 PP, beside overlapping proteins, PRG4, hemoglobin subunit beta (HBB), insulin-like growth factor-binding protein acid-labile subunit (IGFALS), plasma amyloid P-component (APCS), transthyretin (TTR), WAP domain-containing protein (LOC100850808), and matrix Gla protein (MGP) were more abundant in the EFA + CLA group as compared to the CTRL group (Fig. 2E). The PCYOX1 and filamin A (FLNA) were less abundant in EFA + CLA than in CTRL (Fig. 2E, Supplementary S2, Supplementary Table S28). Herein in response to EFA + CLA supplementation, only negative regulation of the immune system process (GO:0002683) was enriched. The GO analysis was not significant for those proteins that were less abundant in the EFA + CLA group than in the CTRL group (Supplementary S8, Supplementary Tables S29, S30, S31).

On day 63 PP, we identified 13 DAP, containing overlapping proteins, coagulation factor XIII A chain (F13A1), C3/C5 convertase (C2), and PRG4 with greater abundance, and LDL receptor-related protein 1 (LRP1), carboxypeptidase A1 (CPA1), adenosylhomocysteinase (AHCY), glyceraldehyde-3-phosphate dehydrogenase (GAPDH), and epoxide hydrolase (EPHX1) with lower abundance in the EFA + CLA group as compared to the CTRL group (Fig. 2F, Supplementary S9, Supplementary Table S32). Again at this timepoint, only overlapping pathways have been enriched (Supplementary S9, Supplementary Tables S33, S34, S35).

## Discussion

This study investigated the plasma proteome of dairy cows supplemented with EFA + CLA during the periparturient period. The DAP and their associated pathways are discussed separately during the transition from late pregnancy to early lactation and between treatment groups. Time-affected DAP will be discussed prior to treatment to gain a deeper understanding of dairy cows' metabolic and immune status while confirming some physiological adaptations that increase the reliability of proteomic data. Then FA supplementations will be discussed as they may affect these metabolic adaptations. Protein–protein interaction network analysis depicts that among DAP identified in this study, some belonged to immune system and lipid metabolic process.

From day − 21 to + 1 of parturition, various immune-related proteins involved in the regulation of pro-inflammatory proteins, such as TNFα, were found to be DAP. This list includes CD14 and adiponectin (ADIPOQ), complement and coagulation cascades such as clusterin (CLU), complement C1q A Chain (C1QA), coagulation factor VII (F7), MASP1, component C2 (C2), CFI, mannose-binding protein C (MBL1), acute-phase response and inflammation comprising haptoglobin (HP), lipopolysaccharide-binding protein (LBP), alpha 2- Heremans-Schmid glycoprotein (AHSG), fibronectin 1 (FN1), and alpha-1-acid glycoprotein 1 (AGP1), and cellular oxidant detoxification protein (GPX3). The involvement of these immune-related proteins is likely due to the activation of the immune system and the development of a systemic inflammation (SI) which is typical in periparturient dairy cows^1^, regardless of their health status, to support a new physiological state.

An excessive SI in early PP is generally associated with massive fat mobilization in conjunction with the release of pro-inflammatory cytokines, TNFα, and interleukin IL6^15–17^ which in turn alter the secretion of acute-phase proteins (APP). Herein, from day − 21 to + 1 relative to parturition and in response to SI, we observed increased abundance of positive APP (HP and LBP) and decreased abundance of negative APP (AHSG and FN1). In addition, AGP1 which is considered a moderate positive APP decreased. Following our results, it is well documented that HP increased from AP to early PP and then gradually decreased^18,19^. In this regard, both HP and LBP are involved in the anti-inflammatory reactions, which are essential to maintain the balanced inflammatory response (for review^1^). Moreover, AHSG, apart from being an APP, is a free fatty acid transporter that enhances cellular lipid uptake and lipogenesis during NEB in dairy cows^20^. Therefore, it seems logical to expect ASHG reduction at the onset of lactation when the lipogenesis pathways were downregulated in favour of energy production. It must be said that the observed AGP1 protein abundances are not in supports of AGP being a positive APP; AGP has been demonstrated to increase during the first and second week of lactation in cows^21^ and water buffalos^22^, respectively. However, there is no comparable study reporting the AGP1 differences between AP and PP in dairy cows. Remarkably, AGP1 has been reported to act as an anti-inflammatory protein^23^, thus being crucial for maintaining immune tolerance. Moreover, it has been reported that both AHSG and AGP1 were reduced before the onset of ketosis in dairy cows^24^. Considering that dairy cows are prone to develop (subclinical) ketosis during the early PP phase, reduced abundance of AHSG and AGP1 in our study is in line with this report.

On the other side, the relative abundance of several complement proteins was reduced at calving. Proteins of the complement system comprise a complex enzymatic cascade that possesses anti-inflammatory functions^25^. The literature on complement proteins in dairy cows plasma, especially during the periparturient period, is scarce. Nevertheless, downregulation of the complement cascade while dairy cows were in SI, implies reduced immune system responsiveness during the early PP period. These results are supported by previous studies that reported immunosuppression in dairy cows before or around calving^26,27^.

During transition from AP to PP, five proteins namely phospholipid transfer protein (PLTP), LRP1, and a cluster of APO containing APOE, APOH, and APOB were annotated by the cholesterol metabolism pathway. The relative abundance of all these proteins, except APOE, was downregulated. In accordance, increased ApoE and decreased ApoB100 mRNA abundance have been reported in the liver of dairy cows during the transition from d − 35 AP to d 3 PP, whereby APOB was correlated with decreased cholesterol plasma levels^28^. Reduced cholesterol synthesis is also a part of the APP response^1^ and is typically observed during early lactation^29^.

Hepatic and whole-body cholesterol homeostasis are regulated by the different lipoprotein classes and their APO particles^3^. Concerning the role of the DAP in cholesterol metabolism, PLTP stimulates phospholipid transfer from very-low-density lipoproteins (VLDL) to the high-density lipoprotein (HDL), and its deficiency is associated with decreased plasma HDL, APOA1, and APOB levels^30^. Also, LRP1 deficiency reduced plasma clearance of APOE-containing lipoproteins^31^ and thereby accelerated hepatic steatosis^32^. In this regard, ApoE which acts as a receptor-binding ligand is highly associated with both VLDL and HDL in the plasma^31^, and its high level leads to the development of lipid-related disorders such as fatty liver in periparturient cows^33^. In addition, decreased ApoB100 is associated with decreased synthesis and secretion of VLDL during the periparturient period. A positive correlation (r = 0.65) has been confirmed between ApoB100 and plasma cholesterol in early PP dairy cows^28^. Furthermore, ApoH reduces the intracellular accumulation of cholesterol and blocks the oxidation of LDL^31^, which in turn inhibits steatosis development.

In line with our proteomics findings, we have previously reported^6^ that the plasma concentration of total cholesterol, LDL-cholesterol, HDL-cholesterol as well as energy balance were at a minimum level on day 1 PP. Decreased APO and lipoproteins suggested reduced cholesterol metabolism (synthesis and reverse transport) and probably initiation of steatosis by the hepatic accumulation of triglycerides (TG) at the onset of lactation.

Interestingly, on day 28 of lactation, some of the immune-related DAP showed the reverse trend compared to d1 PP and tended back to AP levels. In this sense, the APP HP, ITIH4, LBP, and cellular oxidant detoxification protein (PRDX2) were downregulated, and CD14, ADIPOQ, and two proteins related to activation and regulation of the immune system process (HRG, MBL2) were upregulated. Taken together, these observations suggest that the compromised immune system at the beginning of lactation was gradually recovering during the first weeks of lactation. These results support the concept that dairy cows have to adapt to profound metabolic and immune challenges around parturition related to a state of SI. However, they usually recover within few weeks by dampening the inflammatory reaction.

From day 28 to 63 PP, only slight fold changes in protein abundance were observed; the relative abundance of alpha-1-antiproteinase (SERPINA1), alpha-2-macroglobulin (A2M), complement factor B (CFB), protein HP-20 homolog (C1QC) associated with complementing and coagulation cascades were decreased. These results further confirm our previous conclusion that suboptimal immune and metabolic functions are largely limited to early lactation and then gradually improve.

In response to EFA + CLA supplementation as compared to the CTRL group, APOC3, APOA1, and APOA4 had greater abundances at all timepoints. Genes encoding all these proteins have been shown to share a common enhancer sequence, meaning they operate in synergy^34^ probably under the control of transcription factors like PPAR^35^. They are predominantly transcribed, translated, and secreted by hepatocytes in many species including dairy cows^36^. These exchangeable APO have a pivotal role in cholesterol homeostasis as a major component of circulating lipoproteins (chylomicrons, VLDL, LDL, and HDL)^3^ and immune cell function^37^, also exerting anti- and pro-inflammatory effects^38–40^. Considering the differential expression of APO during the periparturient period and between treatments, as well as their involvement in both lipid metabolism and the immune system, this study suggests APO (as well as their associated molecules lipoproteins) as potential mediators for crosstalk between these two systems.

Besides, fat digestion and absorption and the peroxisome proliferator-activated receptor (PPAR) signaling pathway were among the most important KEGG pathways associated with the identified APO. In that sense, previous studies have demonstrated that PPAR^41^ and insulin^42^ are inhibitors of ApoC3 gene expression, whereas FA^43^ induce ApoC3. It has also been shown that a high-fat diet rich in monounsaturated and omega-3 FA reduced APOC3 in human patients with hypertriglyceridemia and/or hyperlipidemia^44^. In this study, we did not observe any changes in the plasma concentration of insulin by treatment; therefore, it may be proposed that the supplemented FA directly or indirectly increased the abundance of APOC3.

Among APO, APOA4 is associated with the formation and secretion of chylomicrons which are an essential component in the transportation of dietary FA from the intestine to the circulation^3^. Also, APOA4 has been shown to regulate TG-rich lipoprotein secretion from mice hepatocytes by stimulating APOB activity and enhancing APOB-containing particle expansion in the secretory pathway, although the underlying mechanisms are not clear^46^. More specifically, studies on human hepatocytes and transgenic mice have shown that APOA1^45^ and APOA4^46^ could reduce hepatic TG accumulation by suppressing endoplasmic reticulum stress and thus restraining the development of steatosis. In this sense, APOA1 is a major component of HDL, and as such involved in bringing cholesterol from peripheral tissues back to the liver (reverse cholesterol transport in dairy cows)^47^. It has been reported that cows with fatty liver and ketosis had lesser serum concentrations of APOA1 which was associated with increased NEFA and decreased cholesterol and phospholipid concentrations^47^. Also, APOC3 is mainly involved in VLDL production and thus it may reduce intracellular TG accumulation^48,49^. Recently, it has been proposed that the downregulation of plasma APOC3 protein in over-conditioned cows is associated with an impaired assembly of VLDL contributing to hepatic TG accumulation^12^.

These results could be a possible explanation for previous observations of specific FA being capable of reducing TG accumulation in cows’ liver^4^. Since the ruminants` ability to export TG as a constituent of VLDL from the liver is limited as compared to non-ruminant species^50^, these APO may accomplish TG transport from the liver. Following this background, the PP concentrations of NEFA in plasma and of TG in the liver were lower in the EFA + CLA than in the CTRL group^6^. Our results support the common concept that APOC3, APOA1, and APOA4 are essential in the metabolism and transport of supplemented FA to the target organs. In this regard, decreased plasma concentrations of APOB-100, APOA1, and APOC3, along with the induced secretion of HP, were reported to be associated with the development of fatty liver^51^. Our results, therefore, suggest that EFA + CLA supplementation was increasing the abundance of proteins that may avoid fatty liver. Our results are in line with previous results stating that CLA supplementation decreased the liver and plasma TG concentrations during the PP period^6^. Moreover, other studies reported that dietary CLA alleviates non-alcoholic fatty liver disease in humans^52^ and rats^53^. On the other hand, there are reports, indicating that CLA supplementation induced fatty liver in mice^54^. This inconsistency can be due to different FA profiles, isomers, and dosage of administrated FA to the animal along with liver physiological differences between species.

Apart from the APOA1–APOC3–APOA4 cluster, a new member of the APO family, APOC4, was found overabundant exclusively in EFA + CLA treated cows when compared to the CTRL cows during the PP period. APOC4 is primarily synthesized in small intestine enterocytes, but its function in hepatocytes is still uncertain^55^. The APOC4 belongs to the APOC family, making another gene cluster together with APOE, APOC1, and APOC2^56^. Both APOC4 and APOE were detected in this study and are reportedly associated mainly with VLDL and TG metabolism in humans^57,58^. As mentioned earlier, the plasma concentration of TG was lower in the EFA + CLA group (data published elsewhere^6^), and these differences were significant on days 14, 28, and 32 PP. There are no reports on APOC4 in cattle, and its roles are yet to be elucidated in dairy cows. Given that APO and probably their associated lipoproteins are increased, it can be concluded that EFA + CLA supplementation elevated the capacity of transferring supplemented FA to the peripheral tissues including the liver, of hepatic TG export, and of reverse cholesterol transport.

At the onset of lactation, coagulation factors 9 and 7, and serum amyloid A (SAA) were more abundant in the EFA + CLA group than in the CTRL group. SAA increases its plasma concentration in cows during the systemic reaction to inflammation^59^, although it is worth mentioning that SAA also plays a role in the reverse cholesterol transport as a minor constituent of HDL, and diseases come secondary after a substantial increase in SAA (inflammation) that displaces APOA1 from HDL and form acute-phase HDL (HDL-SAA) (for review^60^). Increased immune-related proteins with FA supplementation coincide with PP immune suppression and may provide another piece of this complex puzzle of the way FA may induce immune-modulatory effects. However, the exact related pathways yet have to be elucidated.

On day 28 PP, the serum amyloid P component (SAP, also known as APCS) was more abundant in the EFA + CLA than in the CTRL group. The SAP is primarily secreted by hepatocytes and macrophages into the circulation and acts as a soluble pattern recognition receptor of the innate immune system^61^. Database searches and functional enrichment analysis demonstrated that SAP associated with HDL might play a role in cholesterol removal from cells^62^. Furthermore, there is mounting evidence that in mice treated with SAP, the plasma paraoxonase1 (PON1) activity was increased^63^. In this regard, a significantly greater paraoxonase activity level was observed in the EFA + CLA group, albeit limited to day 49^2^.

At the last timepoint (day 63 PP), concurrent with the shift to a positive EB in the EFA + CLA group, no other pathways were detected or activated apart from common APO pathways (overlapping pathways). This suggests that the beneficial effects of supplemented FA were more relevant during physiological and immunological challenges in the early PP phase.

## Conclusion

The plasma protein profiles during the periparturient period provided molecular insight into the systemic inflammatory state, reduced responsiveness of immune response, and impaired cholesterol metabolism at the onset of lactation in dairy cows, which gradually recovered with time PP by reduced their inflammatory response. In addition, this study provides novel knowledge on how EFA + CLA supplementation and the related changes in plasma proteome may act in the crosstalk between lipid metabolism and immune responsiveness in dairy cows. The underlying mechanism might be partly associated with the functions of the APO in regulating hepatic cholesterol and TG metabolism and their emerging role in modulating immune functions in a time-dependent manner. The over-abundance of APOA1–APOC3–APOA4 was explicitly related to EFA + CLA supplementation (regardless of time), although APOC4 was only elevated during lactation. Collectively, our results suggest a beneficial effect of EFA + CLA supplementation on the prevention of hepatic lipid accumulation during the periparturient period. However, future research should integrate proteomics with other results on specific markers of fatty liver to develop reliable metaphylactic and therapeutic strategies.

## Materials and methods

### Animals, treatments, and experimental design

All the experimental procedures were carried entirely under animal welfare guidelines and were approved by the ethics of the State Mecklenburg-Western Pomerania, Germany (LALLF M-V/TSD/7221.3‐1‐038/15). This study was in the frame with a recent comprehensive project described in detail by Vogel et al.^6^. In brief, 16 Holstein dairy cows in their second lactation from 63 days before to 63 days after parturition were abomasally (10 cm center diameter rumen cannula; Bar Diamond Inc., 106 Parma, ID) infused with one of the two treatments, 1: CTRL, (n = 8; coconut oil, Bio-Kokosöl #665, Kräuterhaus Sanct Bernhard, KG, Bad Ditzenbach, Germany; 76 g/day), this supplement was formulated to compensate for energy intake of EFA treatment and provided no EFA and CLA, 2: EFA + CLA, a combination of linseed oil (DERBY Leinöl #4026921003087, DERBY Spezialfutter GmbH, Münster, Germany; 78 g/day), safflower oil (GEFRO Distelöl, GEFRO Reformversand Frommlet KG, Memmingen, Germany; 4 g/day) and Lutalin (CLA, n = 8; cis-9, trans-11, 10 g/day trans-10, cis-12 CLA, BASF SE, Lampertheim, Germany; 38 g/day). During the dry period, each dose was halved. Oil supplements were provided twice daily at 0700 and 1630 h in equal portions through abomasal infusion lines (Teflon tube [i.d. 6 mm] with two perforated Teflon flanges [o.d. 120 mm], directly into abomasum to avoid ruminant degradation of the fatty acids. The cows were housed in free-stall barns with ad libitum access to a corn silage-based total mixed ration (TMR), formulated according to recommendations provided by the Society for Nutrition Physiology (GfE, 2001^64^, 2008^65^, 2009^66^) and Deutsche Landwirtschaftliche Gesellschaft (DLG, 2013), for AP and PP^67^. No clinical signs of disease were observed in any of the dairy cows during the experiment.

Ingredients, the chemical composition of the experimental diets, the amount and FA compositions of lipid supplements, FA composition of the experimental diets, and FA composition of the daily infused supplements during lactation are given in Supplementary S10, Supplementary Tables S36, S37, S38, and S39. The concentrations of NEFA, triglycerides (TG), low-density lipoprotein cholesterol (LDL-C), high-density lipoprotein cholesterol (HDL-C), and total cholesterol (TC) in blood plasma and TG in the liver can be found in Vogel et al.^6^.

### Blood sampling and plasma preparation for proteomics analysis

Blood (about 5 mL) was sampled from each cow on days − 21, + 1, + 28, and + 63 relative to calving. Sampling was performed immediately after the morning milking and before feeding by jugular vein puncture using a Vacuette system (Greiner Bio-One International GmbH, Kremsmünster, Austria) containing K_3_EDTA (1.8 g/L) as an anticoagulant. Immediately after collection, the samples were cooled on crushed ice and centrifuged at 1500×*g* (4 °C, 20 min). The supernatant was harvested and stored at − 80 °C until analysis.

Before protein extraction, plasma samples were thawed slowly in the fridge (2 to 8 °C) and then centrifuged at 10,000×*g* for 10 min at 4 °C to precipitate cells, debris, and aggregated proteins in the bottom. Enrichment of low-abundance plasma proteins was performed using the ProteoMiner small capacity kit (#163-3006, Bio-Rad Laboratories, Inc., CA, USA), according to the manufacturer's instructions.

Before digestion, the protein concentration was measured by the bicinchoninic acid assay (BCA) kit (Thermo Scientific, Rockford, IL, USA) using bovine serum albumin as a reference protein standard. Protein digestion was performed in the S-Trap filter according to the manufacturer’s procedure. Briefly, 100 μg of extracted proteins were first mixed with 4% SDS and 20 mM DTT (final concentrations) and boiled for 10 min at 95 °C. After cooling at RT, proteins were alkylated by adding 50 mM iodoacetamide, followed by incubation in the dark for 45 min. The procedure was followed by acidification of samples to a final concentration of 1.2% phosphoric acid (~ pH 2) for better adhesion to the filter. After that, six volumes of S-Trap binding buffer (90% methanol; 100 mM triethylammonium bicarbonate, TEAB; pH 7.1) were added to the samples. Following a gentle mixing, the protein solution was loaded to a S-Trap filter, centrifuged at 4000*g* for 10 min at RT, and the flow-through collected and reloaded onto the S-trap filter. This step was repeated two times, and then the filter was washed with 150 μL of binding buffer (two times). Finally, protein digestion was performed by adding 2 μg of sequencing-grade trypsin and 150 μL of digestion buffer (50 mM TEAB) to the filter for overnight at 37 °C.

After the digestion step, peptides were eluted in two steps: in the first step, 40 µL of 50 mM TEAB, 0.2% formic acid in H_2_O, and the second step, 50 μL 50% acetonitrile 0.2% formic acid in H_2_O were applied. Before injection, peptides were dried in a Speed Vac (Eppendorf AG, Hamburg, Germany) for 1 h and suspended in 20 µL of equilibration solution (H_2_O/Trifluoroacetic Acid − 99.95/0.05).

### Liquid chromatography-mass spectrometry analysis

The plasma peptide mixture was analyzed by label-free LC–MS/MS quantitative proteomics approach using an Ultimate 3000 RSLCnano system (Thermo Fisher Scientific)) coupled to an Orbitrap Q Exactive HF-X mass spectrometer (Thermo Fisher Scientific) with a Nanospray Flex Ion Source, according to the previously described method by Ref.^68^. Briefly, 1 μL of hydrolyzate was first preconcentrated and desalted at a flow rate of 30 µL/mn on a C18 pre-column 5 cm length X 100 µm (Acclaim PepMap 100 C18, 5 µm, 100A nanoViper) equilibrated with Trifluoroacetic Acid 0.05% in water to remove contaminants that could potentially disrupt the efficiency of the mass spectrometry analysis. After 6 min, the concentration column was switched online with a nano debit analytical C18 column (Acclaim PepMap 100—75 µm inner diameter × 25 cm length; C18—3 µm—100 Å—SN 10711311) operating at 400 nL/min equilibrated with 96% solvent A (%99.5% H_2_O, 0.5% formic acid). The peptides were then separated according to their hydrophobicity, thanks to a gradient of 4 to 20% solvent B (99.5 ACN, 0.5% formic acid) in 60 min.

For MS/MS analysis, eluates were electro-sprayed in positive-ion mode at 1.6 kV through a nanoelectrospray ion source heated to 250 °C. The Orbitrap Q Exactive HF-X MS was used in HCD top 18 mode (i.e. 1 full scan MS and the 18 major peaks in the full scan were selected for MS/MS). Higher-energy C-trap dissociation (HCD): HCD refers to a CID variation that uses a higher RF voltage to retain fragment ions in the C-trap. Mass spectrometry analysis parameters were as follows: the parent ion is selected in the orbitrap cell (FTMS) at a resolution of 60,000 with an injection time of 50 ms on a mass range from 375 to 1600 *m/z*. Each MS analysis is followed by 18 MS/MS with analysis of MSMS fragments at a resolution of 15,000 with an injection time of 100 ms.

### Data processing, statistical analysis, and functional enrichment analysis

Each acquired raw MS/MS spectrum was first aligned to the reference sample (assigned automatically with having the highest of peptide ions coverage) and then processed for peptide ions identification using Progenesis QI software (version 4.2, Nonlinear Dynamics, New Castel upon Tyne, UK), with the default parameter settings (ion charged set to five and Ions ANOVA P-value < 0.05). The identified and the quantified peptide ions were then searched against *Bos taurus* decoy database (Uniprot, download date: 2019/11/07, a total of 37,513 entries) in MASCOT (version 2.5.1) interrogation engine, with the following setting: trypsin as an enzyme digest of a protein, tryptic specificity to cleavage C-terminal after lysine or arginine residues, allowing two missed cleavages, variable modification to carbamidomethylation (C) and oxidation (M), mass tolerance to 10 ppm for precursor ions and 0.02 Da for fragment ions, and FDR < 0.01. Accordingly, the corresponding proteins with at least two peptides and two unique validated peptides were identified and quantified based on their intensities.

Before statistical analysis, logarithmic transformation was applied to protein intensities, and missing intensities (with the frequency of less than 50% of samples) were imputed and replaced by the 1/5 of the minimum positive value of each variable in the original dataset. DAP were identified and investigated separately during the time and between treatments. The time associated DAP were identified using paired t-test between each consecutive time-point to cope with carryover effects.

At each timepoint, the most important proteins (VIP) involved in the discrimination of the CTRL and EFA + CLA groups was identified using Partial Least Square Discriminant Analysis (PLS-DA) analysis (mixOmics package in R). The VIP contributed to the projection scores (> 1.5) of the first two components (PC1 and PC2) and to cluster separation between treatment groups were ranked. A permutation test (100 random computations) was applied to disprove the over-fitting of the PLS-DA model. However, the significance of permutation test is more relevant for a prediction purpose, which is not the goal of our study. The permutation test was not significant at all timepoints and there was a possibility for false positive detection. Therefore, the second filtration step was applied and only VIP with P-value < 0.05, and log2 (fold change) > 1.3 (metaboanalyst R package) were maintained and considered as differentially abundant proteins (DAP) for gene ontology functional enrichment analysis. The P-value was assessed by Student's t-test. Statistical analysis was performed in mixOmics and metaboanalyst R-packages in R statistical software (R version 4.0.0). DAP were visualized according to their expression (by Volcano plot, EnhancedVolcano R package). The GO categorization, including BP, MF, and CC, and KEGG (www.kegg.jp/kegg/kegg1.html)^14^ pathway enrichment analysis were conducted using the web-based tool String version 11.0, summarized in REVIGO web-based tool (http://revigo.irb.hr/) and visualized in Cytoscape software (https://cytoscape.org, version 3.8). The *B. taurus* interaction map was set as a background list, and pathways (false discovery rate < 0.05) with at least two protein hits were considered as enriched pathways. Protein–protein interaction networks (PPI) were constructed by inputting the time and treatment DAP to proteINSIDE (V 2.0) tool. PPI were searched in *Homo sapiens* to take advantage of the well-characterised physical interactions between proteins, and PPI agreed by curator review were used to construct PPI networks (Supplementary Fig. S1). Within the network, proteins that interact to contribute to cellular or metabolic processes have been highlighted using betweeness or closeness centralities as previously described^69^.

### Proteomics data matched the classical measurements of proteins and metabolites

To validate the proteomics results, we integrated the serum proteome data with plasma metabolites and proteins^6,70^ using sparse partial least squares (sPLS) regression model (mixOmics package in R). With X (proteomics data) and Y (classical plasma proteins and metabolites data) as input matrices (logarithmic transformed values), the sPLS model controls many noisy, collinear (correlated) and missing variables in omics datasets, and relates multiple outcome variables across X and Y. Positive and significant correlations were observed between 1—proteomics and ELISA-measured HP; 2—proteomics and Western blot-measured IGFBP2; 3—NEFA with LBP and IGFBP2; 4—LDL with APOB, APOC4, and APOC3 proteins (Supplementary Fig. S2A,B). Although, ADIPOQ ELISA results were not correlated (r = 0.05, P-value = 0.66) with proteomics results. Also, using the bovine AHSG ELISA Kit (Catalog No: DL-aHSG-b, DLdevelop, Wuxi, China), and testing a series of different plasma dilutions, we could not detect AHSG in our plasma samples. This was probably attributable to an inadequate binding specificity of the antibody used in the AHSG ELISA kit. Additionally, despite the accuracies of the mass spectrometry and immunological assays that we classically used to quantify protein abundances, methodological specificities may provide some differences in the abundances assayed.

### Ethics declaration

All the experimental procedures were carried entirely under animal welfare guidelines (including ARRIVE guidelines) and were approved by the ethics of the State Mecklenburg-Western Pomerania, Germany (LALLF M-V/TSD/7221.3‐1‐038/15).

## Supplementary Information

Supplementary Tables S1–S2.

Supplementary Tables S3–S8.

Supplementary Tables S9–S14.

Supplementary Tables S15–S18.

Supplementary Table S19.

Supplementary Tables S20–S23.

Supplementary Tables S24–S27.

Supplementary Tables S28–S31.

Supplementary Tables S32–S35.

Supplementary Tables S36–S39.

Supplementary Figure S1.

Supplementary Figure S2.

Supplementary Information.

## Data Availability

The data and the related analyses are available through the link 10.15454/ZIIL2H.
