# Supplementary material for: Plasma proteomics reveals crosstalk between lipid metabolism and immunity in dairy cows receiving essential fatty acids and conjugated linoleic acid
Source: Sci Rep. 2022 Apr 5;12:5648. doi: 10.1038/s41598-022-09437-w (PMC8983735; doi:10.1038/s41598-022-09437-w)
Supplement: Supplementary file 10 — Supplementary Tables S36–S39. [file 41598_2022_9437_MOESM10_ESM.docx]

**Supplementary S10 (extracted from Vogel et al., 2020)**

Supplementary S10 Tables S36. Amounts of daily (abomasally) infused supplements^1^.

| Supplementation | treatment | | | | |
| --- | --- | --- | --- | --- | --- |
|  | CTRL^2^ |  | EFA+CLA | | |
|  |  |  | Linseed oil^3^ | Safflower oil^4^ | Lutalin®^5^ |
| Daily infused oils (g/d) |  |  |  |  |  |
| Dosage lactation | 76 |  | 78 | 4 | 38 |
| Dosage dry period | 38 |  | 39 | 2 | 19 |
| Daily infused fatty acids (g/d) at the lactation dosage^6^ |  |  |  |  |  |
| 18:3 cis-9, cis-12, cis-15 | 0.00 |  | 39.9 | 0.01 | 0.00 |
| 18:2 cis-9, cis-12 | 1.39 |  | 12.4 | 2.48 | 1.34 |
| 18:2 cis-9, trans-11 | 0.00 |  | 0.00 | 0.01 | 10.3 |
| 18:2 trans-10, cis-12 | 0.00 |  | 0.02 | 0.01 | 10.2 |

^1^Cows were supplemented daily with coconut oil (CTRL), or mixture of linseed, safflower oil (EFA), and Lutalin® (CLA, c9, t11 and t10, c12), (EFA+CLA).

^2^Addition of vitamin E (0.06 g/d), Covitol 1360 (BASF, Ludwigshafen, Germany), to compensate for the vitamin E in linseed oil (0.07%) and

safflower oil (0.035%).

^3^DERBY, Derby Spezialfutter GmbH, Münster, Germany

^4^GEFRO, Memmingen/Allgäu, Germany

^5^BASF, Ludwigshafen, Germany

^6^The lactation dosage was halved during the dry period.

Supplementary S10 Table S37. Ingredients and chemical compositions of the diets.

| Item (g/kg of DM) | Diet | | |
| --- | --- | --- | --- |
|  | Dry period^1^ | Lactation | |
| Ingredients | 421 | 457 |  |
| Corn silage  Straw | 223 | 97 |  |
| Compound feed DEFA^2^ (granulated) | - | 446 |  |
| Dried sugar beet pulp | 163 | - |  |
| Extracted soybean meal | 99 | - |  |
| Grain of rye | 75 | - |  |
| Mineral-vitamin mixture^3^ | 10 | - |  |
| Urea^4^ | 9 | - |  |
| Chemical composition |  |  |  |
| NEL5 (MJ/kg DM)^6^ | 6.2 | 7.1 |  |
| Crude fat | 21 | 23 |  |
| Crude fiber | 219 | 173 |  |
| Crude protein | 141 | 146 |  |
| Utilizable protein^6^ | 141 | 143 |  |
| NFC^7^ | 379 | 432 |  |
| NDF^8^ | 423 | 346 |  |
| ADF^9^ | 249 | 197 |  |
| RNB^6,10^ | 0.0 | 0.5 |  |

^1^ The dry period diet was fed from wk 6 to wk 1 before calving.

^2^ Ceravis AG, Malchin, Germany Ingredients: 46.5% dried sugar beet pulp, 25.3% extracted soybean meal, 23.8% grain of rye, 1.4% urea, 1.1% premix cow, 1.00% calcium, 0.37% phosphorus, 0.42% sodium, vitamins A, D3, E, copper, ferric, zinc, manganese, cobalt, iodine, selenium Chemical composition: 44.4% NFC, 24.1% crude protein, 21.6% NDF, 12.4% ADF, 9.3% crude fiber, 8.2% crude ash, 1.8% crude fat, 7.9 MJ NEL/kg DM

^3^ KULMIN®MFV Plus (Bergophor Futtermittelfabrik Dr. Berger GmbH & Co. KG, Kulmbach, Germany): 8.5% magnesium, 7.5% phosphorus, 6.5% sodium, 3.5% HClinsoluble ash, 1.5% calcium, additives: vitamins A, D3, E, B1, B2, B6, B5, B3, B12, B9, H, zinc, manganese, copper, cobalt, iodine, selenium, and Saccharomyces cerevisiae

^4^ Piarumin® (SKW Stickstoffwerke Piesteritz GmbH, Lutherstadt Wittenberg, Germany): 99% urea, 46.5% total nitrogen

^5^ NEL= net energy for lactation

^6^ German Society of Nutrition Physiology (2001, 2008, 2009) and Deutsche Landwirtschaftliche Gesellschaft (DLG, 2013)

^7^ NFC= nonfibrous carbohydrates (NFC)

^8^ NDF= neutral detergent fiber (NDF)

^9^ ADF= acid detergent fiber

^10^ RNB = ruminal nitrogen balance

Supplementary S10 Table S38. Fatty acid composition of the experimental diets.

|  | Diet | |
| --- | --- | --- |
| Fatty acid (g/kg of DM) | Lactation | Dry period1 |
| 10:0 | 0.01 | 0.01 |
| 12:0 | 0.04 | 0.03 |
| 14:0 | 0.12 | 0.18 |
| 15:0 | 0.04 | 0.04 |
| 16:0 | 4.73 | 4.53 |
| 16:1, cis-9 | 0.06 | 0.05 |
| 17:0 | 0.09 | 0.08 |
| 17:1, cis-9 | 0.01 | 0.01 |
| 18:0 | 0.63 | 0.60 |
| 18:1, cis-9 | 4.82 | 3.84 |
| 18:1, cis-11 | 0.28 | 0.21 |
| 18:2, cis-9, cis-12 | 9.63 | 9.32 |
| 18:3, cis-9, cis-12, cis-15 | 1.35 | 1.37 |
| 18:4, cis-6, cis-9, cis-12, cis-15 | 0.04 | 0.02 |
| 20:0 | 0.15 | 0.16 |
| 20:1, cis-11 | 0.08 | 0.06 |
| 20:2, cis-11, cis-14 | 0.05 | 0.02 |
| 21:0 | 0.01 | 0.02 |
| 22:0 | 0.18 | 0.25 |
| 22:1, cis-13 | 0.01 | − |
| 22:2, cis-13, cis-16 | 0.01 | 0.04 |
| 23:0 | 0.05 | 0.02 |
| 24:0 | 0.23 | 0.29 |
| SFA2 | 6.27 | 6.21 |
| MUFA3 | 5.27 | 4.17 |
| PUFA4 | 11.08 | 10.77 |
| Sum of n-3 fatty acids5 | 1.39 | 1.39 |
| Sum of n-6 fatty acids6 | 9.69 | 9.38 |
| Ratio of n-6/n-3 | 7.00 | 6.76 |

^1^The dry period diet was fed from wk 6 to 0 before calving.

^2^Sum of 10:0; 12:0; 14:0; 15:0; 16:0; 17:0; 18:0; 20:0; 21:0; 22:0; 23:0 and 24:0

^3^Sum of 16:1 cis-9; 17:1 cis-9; 18:1 cis-9; 18:1 cis-11; 20:1 cis-11 and 22:1 cis-13

^4^Sum of 18:2 cis-9, cis-12; 18:3 cis-9, cis-12, cis-15; 18:4 cis-6, cis-9, cis-12, cis-15; 20:2 cis-11, cis-14 and 22:2 cis-13, cis-16

^5^Sum of 18:3 cis-9, cis-12, cis-15 and 18:4 cis-6, cis-9, cis-12, cis-15

^6^Sum of 18:2 cis-9, cis-12; 20:2, cis-11, cis-14 and 22:2 cis-13, cis-16

Supplementary S10 Table S39. Fatty acid composition of the daily infused supplements during lactation^1^

|  | CTRL2 |  |  | EFA+CLA3 |
| --- | --- | --- | --- | --- |
| Fatty acid (%) | Coconut oil |  |  | Total |
| SFA | 89.4 |  |  | 10.7 |
| 6:0 | 0.93 |  |  | − |
| 8:0 | 9.85 |  |  | − |
| 10:0 | 6.10 |  |  | − |
| 12:0 | 45.5 |  |  | − |
| 14:0 | 16.9 |  |  | 0.02 |
| 16:0 | 6.87 |  |  | 5.98 |
| 17:0 | − |  |  | 0.02 |
| 18:0 | 3.10 |  |  | 4.13 |
| 20:0 | 0.11 |  |  | 0.21 |
| 22:0 | − |  |  | 0.21 |
| 24:0 | − |  |  | 0.10 |
| MUFA | 8.35 |  |  | 24.1 |
| 16:1, cis-9 | − |  |  | 0.08 |
| 18:1, cis-9 | 8.35 |  |  | 23.1 |
| 18:1, cis-11 | − |  |  | 0.63 |
| 20:1, cis-11 | − |  |  | 0.27 |
| 24:1, cis-15 | − |  |  | < 0.01 |
| PUFA | 1.83 |  |  | 46.9 |
| 18:2, cis-9, cis-12 | 1.83 |  |  | 13.5 |
| 18:2, cis-9, trans-12 | − |  |  | 0.03 |
| 18:2, trans-9, cis-12 | − |  |  | 0.02 |
| 18:3, cis-6, cis-9, cis-12 | − |  |  | 0.02 |
| 18:3, cis-9, cis-12, cis-15 | − |  |  | 33.3 |
| 20:2, cis-11, cis-14 | − |  |  | 0.04 |
| 20:3, cis-11, cis-14, cis-17 | − |  |  | 0.02 |
| 22:5, cis-7, cis-10, cis-13, cis-16, cis-19 | − |  |  | 0.01 |
| CLA | < 0.05 |  |  | 17.2 |
| 18:2, cis-9, trans-11 CLA | − |  |  | 8.61 |
| 18:2, trans-10, cis-12 CLA | − |  |  | 8.56 |
| 18:2, cis-9, cis-11 CLA | − |  |  | 0.07 |

^1^Dosage of oil supplementation was halved during the dry period in all groups, respectively.

^2^Control (CTRL, n=9): 76 g/d coconut oil (Bio-Kokosöl #665, Kräuterhaus Sanct Bernhard KG, Bad Ditzenbach, Germany) and 0.06 g/d Vitamin E (Covitol®1360, BASF SE, Ludwigshafen, Germany).

^3^Essential fatty acids and conjugated linoleic acid (EFA+CLA, n = 8): 78 g/d linseed (DERBY® Leinöl #4026921003087, DERBY Spezialfutter GmbH, Münster, Germany),4 g/d safflower oil (GEFRO Distelöl, GEFRO Reformversand Frommlet KG, Memmingen, Germany) and 38 g/d Lutalin® (BASF SE, Ludwigshafen, Germany).
