# Supplementary material for: Plasma proteomics reveals crosstalk between lipid metabolism and immunity in dairy cows receiving essential fatty acids and conjugated linoleic acid
Source: Sci Rep. 2022 Apr 5;12:5648. doi: 10.1038/s41598-022-09437-w (PMC8983735; doi:10.1038/s41598-022-09437-w)
Supplement: Supplementary file 11 — Supplementary Figure S1. [file 41598_2022_9437_MOESM11_ESM.docx]

Supplementary Figure S1


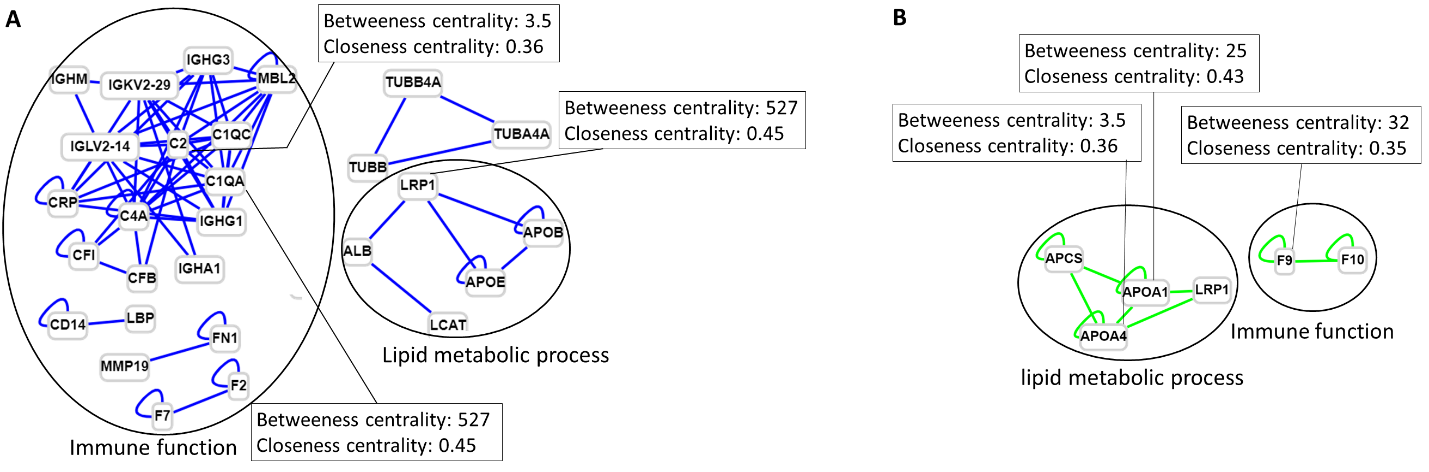


Supplementary Figure S1. Protein−protein interaction network analysis. Networks were constructed by the ProteINSIDE (V 2.0) too. Nodes were DAP identified during the time (A) or between CTRL and EFA+CLA groups (B), and edges were the PPI agreed by curator review in Human species. PPI network were filtered according to betweeness (higher than 0.1, that quantifies how frequently a node is on the shortest path between every pair of nodes for detecting bottlenecks in a network) or closeness (0.1, that quantifies how short are minimal paths from a given node to all others, a large closeness indicates that a node is close to the topological centre of the network) centralities in order to reveal key proteins that play important roles in a network and for a biological pathway.
