# Supplementary material for: Plasma proteomics reveals crosstalk between lipid metabolism and immunity in dairy cows receiving essential fatty acids and conjugated linoleic acid
Source: Sci Rep. 2022 Apr 5;12:5648. doi: 10.1038/s41598-022-09437-w (PMC8983735; doi:10.1038/s41598-022-09437-w)
Supplement: Supplementary file 12 — Supplementary Figure S2. [file 41598_2022_9437_MOESM12_ESM.docx]

Supplementary Figure S2 A.


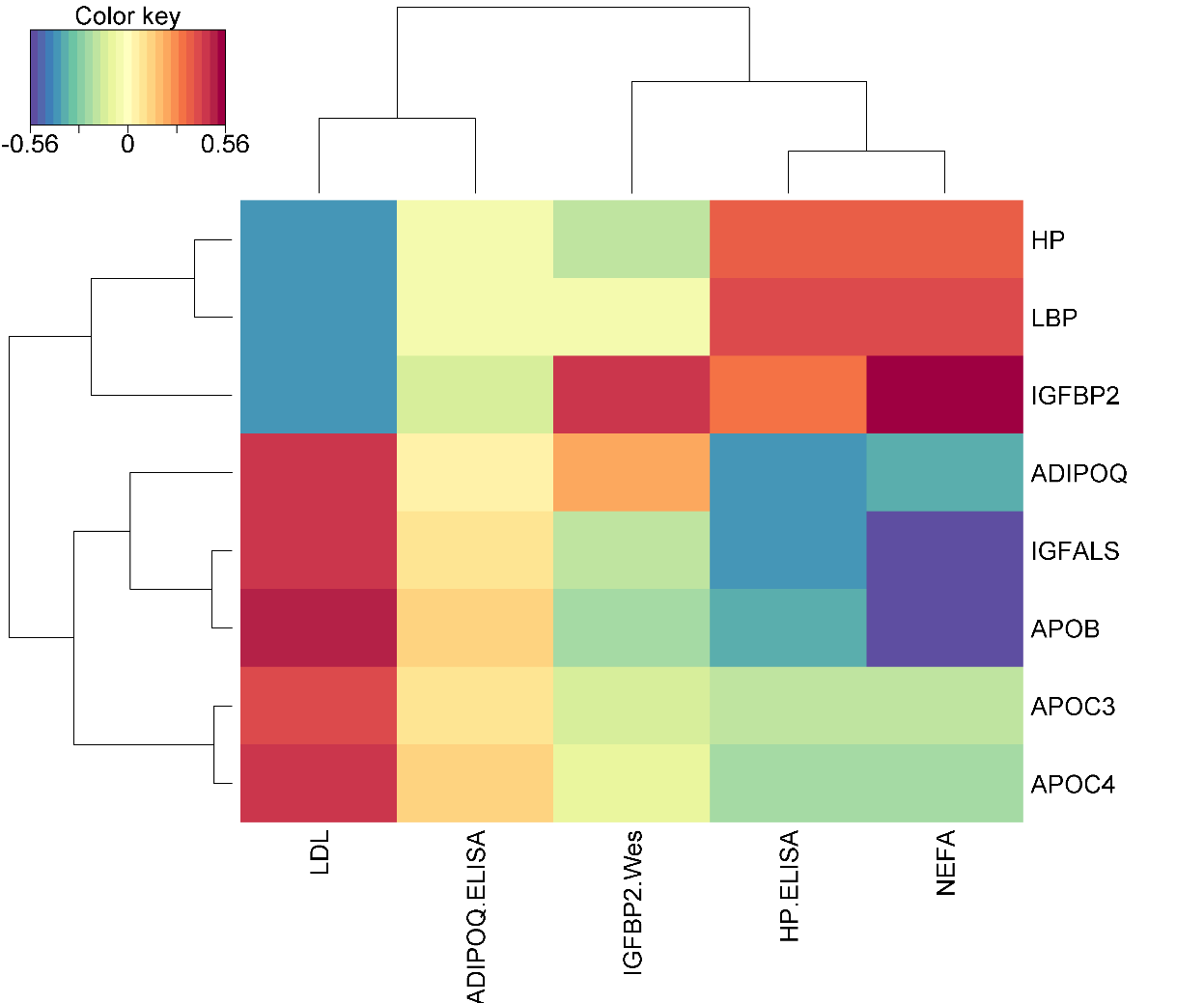


Supplementary Figure S2 B.


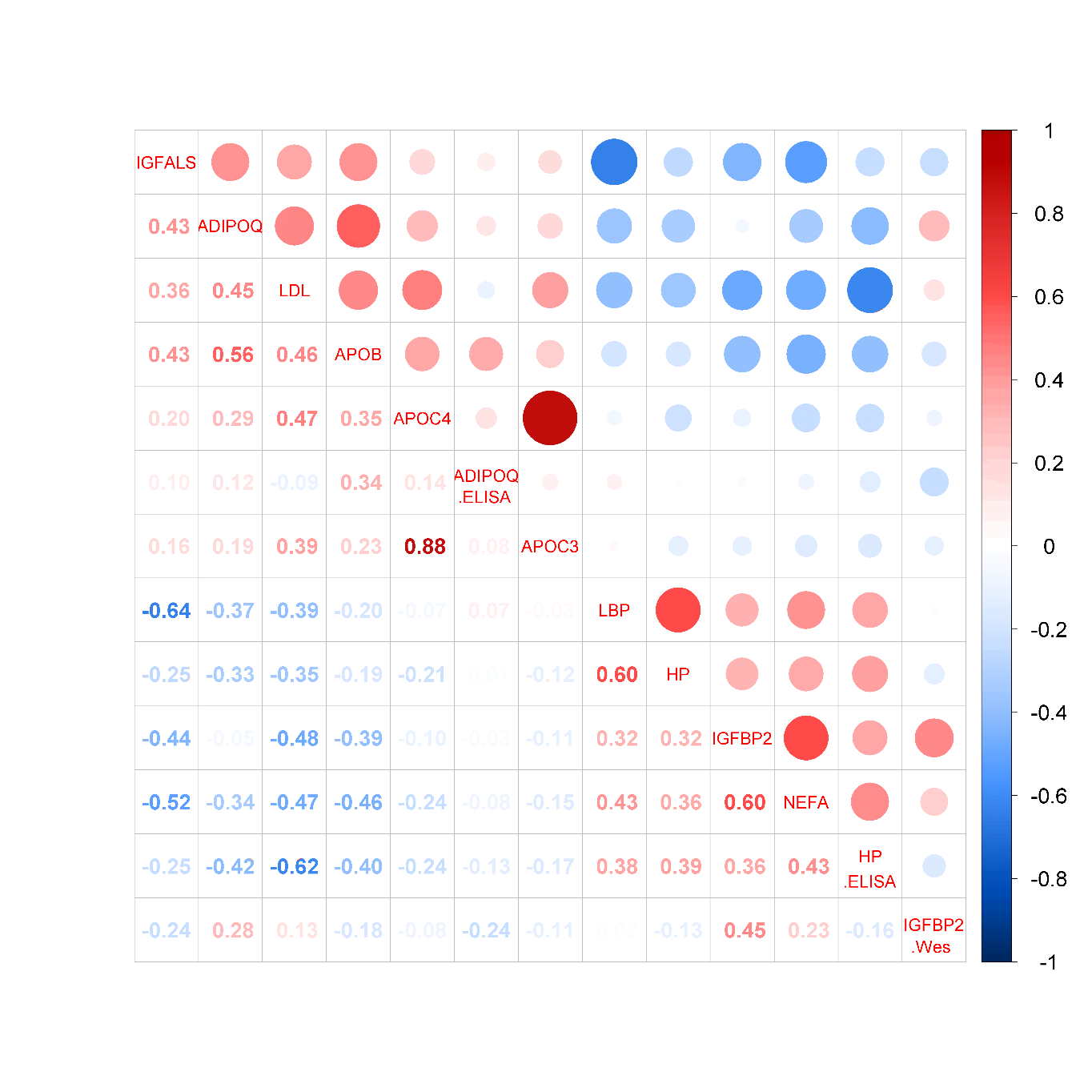


Supplementary Figure S2. A. Clustered image map of integrated plasma proteins (Y-axis) with metabolites and proteins (ELISA and Western blot, X-axis) using sparse partial least squares (sPLS) model. The correlations between the plasma proteins (proteomics-based) and the metabolite and proteins (ELISA and Western blot-measured) by a color gradient on a two-dimensional colored image. The negatively correlated variables (blue) are represented along the positively correlated variables (red). Dendrograms are added to represent the clusters produced by the hierarchical clustering. Partial Least Squares (PLS) regression is a multivariate methodology which relates two data matrices X and Y, and model multiple outcome variables. B. Pearson correlation of proteomics plasma proteins with metabolites and proteins (ELISA and Western blot). The size and color of the circle represent the correlation coefficient value and whether the correlation is negative (blue) or positive (red), respectively. WB= Western blotting. Plasma IGF1, HP, and ADIPOQ were measured using a bovine-specific ELISA. Concentrations of plasma IGFBP2 were analyzed via quantitative Western ligand Blot analysis. Plasma metabolites were analyzed using an automatic spectrophotometer (ABX Pentra 400; HORIBA ABX SAS, Montpellier, France) and respective kits: #434 91795 (NEFA; acyl-CoA synthetase – acyl- CoAoidase method) from WAKO Chemicals GmbH (Neuss, Germany); #A11A01640 (TG; lipoproteinlipase – glycerinkinase – glycerin-phosphate-oxidase method), #A11A01638 (LDL-C; direct measurement of cholesterol in LDL by the cholesterinesterase and cholesterinoxiase and LDL cleavage), and #A11A01636 (HDL-C; direct measurement of cholesterol in HDL by accelerator selective detergent method with cholesterinesterase) from HORIBA ABX SAS (Montpellier, France), and #553-126 (TC; cholesterinoxidase method) from mti-diagnostics GmbH (Idstein, Germany). The concentrations of plasma insulin (#RIA-1257) and glucagon (#RIA-1258) were determined via RIA using kits from DRG Instruments GmbH (Marburg, Germany) ^2,6,7,69^.
