# Supplementary material for: Plasma proteomics reveals crosstalk between lipid metabolism and immunity in dairy cows receiving essential fatty acids and conjugated linoleic acid
Source: Sci Rep. 2022 Apr 5;12:5648. doi: 10.1038/s41598-022-09437-w (PMC8983735; doi:10.1038/s41598-022-09437-w)
Supplement: Supplementary file 13 — Supplementary Information. [file 41598_2022_9437_MOESM13_ESM.docx]

**Supplementary files:**

Supplementary files (.xlsx) were deposited at the INRAe portal as “Gene ontology and functional enrichment analysis of plasma differentially abundant proteins during the transition to lactation and between different fatty acid treatments in Holstein cows”. Here is the Private link:

<https://data.inrae.fr/privateurl.xhtml?token=76505b52-30c0-4e7f-ae2d-5585520bda31>

**1- In the file named ‘S1. proteins accession, gene name, and proteins classes.xlsx’, the reader will find the following info,**

first sheet: Table S1. identified proteins, associated gene names, and peptides info.

second sheet: table S2. Protein class by PANTHER.

**2- In the file named ‘S2. +1d VS -21d DAP and GO analysis.xlsx’, the reader will find the following info,**

first sheet: table S3. Differential plasma proteome during transition from d -21 to +1 relative to parturition.

second sheet: Table S4. KEGG pathways associated to differentially abundant proteins during transition from d -21 to +1 relative to parturition.

third sheet: Table S5. Enriched terms for biological processes of underabundant plasma proteins during transition from d -21 to +1 relative to parturition

fourth sheet: Table S6. Enriched terms for biological processes of overabundant plasma proteins during transition from d -21 to +1 relative to parturition

fifth sheet: Table S7. Enriched terms for cellular components of underabundant plasma proteins during transition from d -21 to +1 relative to parturition

sixth sheet: Table S8. Enriched terms for cellular components of overabundant plasma proteins during transition from d -21 to +1 relative to parturition

**3- In the file named ‘S3. +28 VS +1d DAP and GO analysis.xlsx’, the reader will find the following info,**

first sheet: table S9. Differential plasma proteome during transition from d +1 to +28 relative to parturition.

second sheet: Table S10. KEGG pathways associated to differentially abundant proteins during transition from d +1 to +28 relative to parturition.

third sheet: Table S11. Enriched terms for biological processes of underabundant plasma proteins during transition from d +1 to +28 relative to parturition

fourth sheet: Table S12. Enriched terms for biological processes of overabundant plasma proteins during transition from d +1 to +28 relative to parturition

fifth sheet: Table S13. Enriched terms for cellular components of underabundant plasma proteins during transition from d +1 to +28 relative to parturition

sixth sheet: Table S14. Enriched terms for cellular components of overabundant plasma proteins during transition from d +1 to +28 relative to parturition

**4- In the file named ‘S4. +63 VS +28d DAP and GO analysis.xlsx’, the reader will find the following info,**

first sheet: table S15. Differential plasma proteome during transition from d +28 to +63 relative to parturition.

second sheet: Table S16. KEGG pathways associated to differentially abundant proteins during transition from d +28 to +63 relative to parturition.

third sheet: Table S17. Enriched terms for biological processes of underabundant plasma proteins during transition from d +28 to +63 relative to parturition.

fourth sheet: Table S18. Enriched terms for cellular components of underabundant plasma proteins during transition from d +28 to +63 relative to parturition.

**5- In the file named ‘S5. Venn diagram details’, the reader will find the following info,**

first sheet: Table S19. Venn diagram representation the overlap between differentially abundant proteins (DAP) in response to EFA+CLA supplementation.

**6- In the file named ‘S6. day -21 GO and KEGG.xlsx’, the reader will find the following info,**

first sheet: table S20. Differential plasma proteome between CTRL and EFA+CLA at d -21 relative to parturition.

second sheet: Table S21. KEGG pathways associated to differentially abundant proteins between CTRL and EFA+CLA at d -21 relative to parturition.

third sheet: Table S22. Enriched terms for biological processes of overabundant plasma proteins between CTRL and EFA+CLA at d -21 relative to parturition.

fourth sheet: Table S23. Enriched terms for cellular components of overabundant plasma proteins between CTRL and EFA+CLA at d -21 relative to parturition.

**7- In the file named ‘S7. day 1 GO and KEGG.xlsx’, the reader will find the following info,**

first sheet: table S24. Differential plasma proteome between CTRL and EFA+CLA at d +1 relative to parturition.

second sheet: Table S25. KEGG pathways associated to differentially abundant proteins between CTRL and EFA+CLA at d +1 relative to parturition.

third sheet: Table S26. Enriched terms for biological processes of overabundant plasma proteins between CTRL and EFA+CLA at d +1 relative to parturition.

fourth sheet: Table S27. Enriched terms for cellular components of overabundant plasma proteins between CTRL and EFA+CLA at d +1 relative to parturition.

**8- In the file named ‘S8. day 28 GO and KEGG.xlsx’, the reader will find the following info,**

first sheet: table S28. Differential plasma proteome between CTRL and EFA+CLA at d +28 relative to parturition.

second sheet: Table S29. KEGG pathways associated to differentially abundant proteins between CTRL and EFA+CLA at d +28 relative to parturition.

third sheet: Table S30. Enriched terms for biological processes of overabundant plasma proteins between CTRL and EFA+CLA at d +28 relative to parturition.

fourth sheet: Table S31. Enriched terms for cellular components of overabundant plasma proteins between CTRL and EFA+CLA at d +28 relative to parturition.

**9- In the file named ‘S9. day 63 GO and KEGG.xlsx’, the reader will find the following info,**

first sheet: table S32. Differential plasma proteome between CTRL and EFA+CLA at d +63 relative to parturition.

second sheet: Table S33. KEGG pathways associated to differentially abundant proteins between CTRL and EFA+CLA at d +63 relative to parturition.

third sheet: Table S34. Enriched terms for biological processes of overabundant plasma proteins between CTRL and EFA+CLA at d +63 relative to parturition.

fourth sheet: Table S35. Enriched terms for cellular components of overabundant plasma proteins between CTRL and EFA+CLA at d +63 relative to parturition.

**S10- In the file named ‘S10. diet ingrediants and treatments FA profile.doc’, the reader will find the following info,**

Table S36. Amounts of daily (abomasally) infused supplements.

Table S37. Ingredients and chemical compositions of the diets.

Table S38. Fatty acid composition of the experimental diets.

Table S39. Fatty acid composition of the daily infused supplements during lactation.

**S11- Supplementary Figure S1**

**S12- Supplementary Figure S2**
